# Using Machine Learning Algorithms to Determine the Post-COVID State of a Person by Their Rhythmogram

**DOI:** 10.3390/s23115272

**Published:** 2023-06-01

**Authors:** Sergey V. Stasenko, Andrey V. Kovalchuk, Evgeny V. Eremin, Olga V. Drugova, Natalya V. Zarechnova, Maria M. Tsirkova, Sergey A. Permyakov, Sergey B. Parin, Sofia A. Polevaya

**Affiliations:** 1 Neurotechnology Department, Institute of Biology and Biomedicine, Lobachevsky State University of Nizhny Novgorod, 603022 Nizhny Novgorod, Russia; 2 Laboratory of Autowave Processes, Institute of Applied Physics, Russian Academy of Sciences, 603950 Nizhny Novgorod, Russia; aka.xzib1t@gmail.com; 3 Faculty of Social Sciences, Lobachevsky State University of Nizhny Novgorod, 603022 Nizhny Novgorod, Russia; eugenevc@gmail.com (E.V.E.); permyakov@fsn.unn.ru (S.A.P.); parins@mail.ru (S.B.P.); sofia.polevaia@fsn.unn.ru (S.A.P.); 4 Department of Medical Biophysics, Privolzhsky Research Medical University, 603005 Nizhny Novgorod, Russia; olgadrugova@gmail.com; 5 GBUZ NO “Nizhny Novgorod Regional Clinical Oncological Dispensary”, 603126 Nizhny Novgorod, Russia; nvzar@mail.ru; 6 Clinical Hospital No. 2, Privolzhsky District Medical Center, 603032 Nizhny Novgorod, Russia; cirkova_mariya@mail.ru

**Keywords:** machine learning algorithms, electrocardiogram, post-COVID state, COVID-19, data analysis

## Abstract

This study introduces a novel method for detecting the post-COVID state using ECG data. By leveraging a convolutional neural network, we identify “cardiospikes” present in the ECG data of individuals who have experienced a COVID-19 infection. With a test sample, we achieve an 87 percent accuracy in detecting these cardiospikes. Importantly, our research demonstrates that these observed cardiospikes are not artifacts of hardware–software signal distortions, but rather possess an inherent nature, indicating their potential as markers for COVID-specific modes of heart rhythm regulation. Additionally, we conduct blood parameter measurements on recovered COVID-19 patients and construct corresponding profiles. These findings contribute to the field of remote screening using mobile devices and heart rate telemetry for diagnosing and monitoring COVID-19.

## 1. Introduction

Currently, several viruses have had significant socio-economic and medical impacts, including SARS, A/H1N1, H5N1/H7N9, MERS, Ebola, and the most widespread and impactful of them all, SARS-CoV-2 [1,2]. SARS-CoV-2 is responsible for causing COVID-19, a potentially severe acute respiratory infection that reached pandemic status in 2020. COVID-19 can manifest as mild or severe, affecting various organs [3,4].

Common symptoms of COVID-19 include fever, fatigue, dry cough, loss of smell, and loss of taste [5,6]. Most infected individuals experience mild or asymptomatic cases with spontaneous recovery [2,7]. However, a portion of cases can progress to severe forms requiring oxygen therapy, with some patients becoming critically ill [8]. COVID-19 can trigger intense inflammation, known as a cytokine storm, leading to fatal pneumonia and acute respiratory distress syndrome [9]. The disease can also have long-term systemic effects on cardiovascular health and other complications, such as acute heart failure, renal failure, septic shock, and cognitive impairment [10,11].

SARS-CoV-2 can induce neurotropic manifestations and damage the central nervous system, causing immune cell infections, encephalitis, encephalopathy, and demyelination [12]. Cognitive problems associated with COVID-19 include difficulty concentrating, motor activity reduction, impaired coordination, loss of smell/taste, decreased sensitivity, and cognitive decline [13,14]. Post-COVID syndrome, experienced by a percentage of patients, presents a wide range of symptoms that can persist for several weeks or longer, including weakness, shortness of breath, headaches, joint pains, cognitive impairment, gastrointestinal issues, and more [15].

The causes of post-COVID syndrome are still not fully understood, and various hypotheses are being explored, including direct damage to cells and organs, persistence of the virus in the body, and autoimmune reactions [16,17,18,19,20,21,22,23,24,25,26,27,28,29,30].

These findings highlight the wide-ranging impacts of SARS-CoV-2 and COVID-19 on human health, necessitating ongoing research and care for affected individuals.

Post-COVID syndrome remains challenging to determine, given the involvement of various organ systems and disorders [31]. Mass spectrometric analysis of blood samples has been used to evaluate inflammatory reactions by assessing 96 proteins [32]. Rapid diagnostic methods with high throughput are crucial for timely treatment and rehabilitation of COVID-19 patients [33,34,35,36,37,38,39,40,41,42,43].

Studies have shown that abnormal ECGs and elevated troponin levels at admission are associated with major adverse events in COVID-19 patients [33]. ECG abnormalities are independent of pulmonary infection severity, and reflect various cardiovascular complications [36]. ST segment alteration and signs of left ventricular hypertrophy are associated with worse prognosis, while an abnormal T wave or the presence of ST segment elevation/depression can indicate COVID-19 patient mortality [34,37].

Deep learning models, such as ECGConvnet, have shown promise in distinguishing COVID-19 from other cardiovascular diseases with high accuracy [38]. One-dimensional convolutional neural networks (1D-CNN) have been used to automatically detect COVID-19 using ECG signals [39].

Heart rate sensors on wearable devices have been utilized to identify potential early indicators of COVID-19 symptoms, such as elevated resting heart rate and altered HR/step measurements [40]. HRV analysis using smartphone cameras and wrist-worn devices has shown differences in HRV indicators for some patients during different phases of COVID-19 [41].

In patients with hypoxic respiratory failure, a study found a significant drop in SDNN (a measure of heart rate variability), accompanied by a substantial increase in CRP levels within 72 h, indicating systemic inflammation [42,43].

Cardiac rhythmography, initially limited to stationary registration conditions, has evolved with advancements in telemetric systems, enabling assessment during various natural activities [44,45,46,47].

Convolutional neural networks (CNNs) have emerged as powerful tools for ECG analysis, demonstrating high accuracy in automating heartbeat classification and arrhythmia detection [48,49]. These CNN-based models have shown superior performance, comparable to cardiologist-level expertise, in accurately identifying and categorizing arrhythmias [48,49]. Deep learning approaches, including CNNs, have also shown potential in automating complex cardiac analyses beyond ECG interpretation [50,51].

Remote assessment of human functional state during various activities has been made possible through different methods and technologies [52,53,54,55,56].

In the context of COVID-19, remote screening using mobile devices and heart rate telemetry has shown promise in diagnosing and monitoring the disease. Studies have observed “cardiospikes” in the rhythmogram records of COVID-19 patients, potentially serving as markers for the disease [57].

In this article, we propose a new method for detecting the post-COVID state using ECG data. The method is based on the detection of “cardiospikes” observed in the ECG data of patients who have had a COVID-19 infection. We utilize a convolutional neural network, which has proven itself as an effective method for analyzing ECG data. The detection accuracy in the test sample was 87 percent. Furthermore, we demonstrate that the observed cardiospikes are not the result of hardware–software signal distortions, but have an endogenous nature, making them potential markers for COVID-specific modes of heart rhythm regulation. Additionally, we measured the blood parameters of patients who had recovered from COVID-19 infection and constructed corresponding profiles.

The structure of this work is as follows. In Section 2, we provide a detailed explanation of the ECG data recording method, including the number of records and their organization. We also outline the algorithms used for data analysis and neural network training. Moving on to Section 3, we present the key findings of our study, which include the visual representation of a “cardiospike” the verification process for identifying these spikes, the blood parameter profiles of individuals who have recovered from a COVID-19 infection, and the outcomes of training a neural network to detect cardiospikes in ECG recordings. In Section 4, we delve into a discussion of our results, and propose potential avenues for further research. Finally, we summarize the study’s outcomes in Section 5.

## 2. Methods

**Figure 1:**
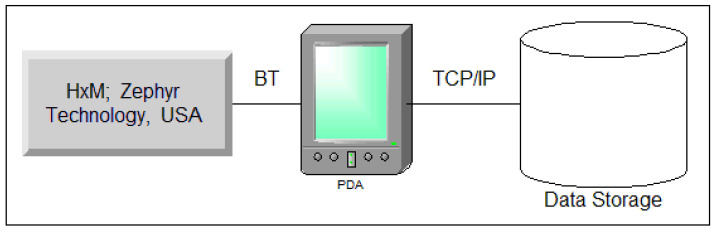
Illustration depicting the setup for recording and analyzing the cardiac rhythmogram system.

A schematic diagram of the cardiac rhythmogram recording and analysis system is depicted in Figure 1.

Event-related heart rate telemetry technology offers several advantages, including mobility (allowing subjects to move freely while reliably recording the signal from a significant distance), uninterrupted recording, autonomous measurement, resistance to external interference, and real-time data acquisition and processing.

To achieve performance goals, we used the ZephyrTM HxMTM Smart-Zephyr BIO PACH BH3-M1 (HxM, Zephyr Technology, Annapolis, MD, USA) sensor platform, including a microprocessor; radio signal receiver/transmitter; and ECG, acceleration, and distance sensors.

Data were transmitted to a smartphone/PC via Bluetooth SPP-2.4 GHz channel at 1-second intervals (Figure 1). Each packet includes sensor platform ID, last 15 R-R intervals, and time relative to recording start. Data is pre-processed on the Android smartphone, then transmitted to a server via GSM channels. Experimental data can be exported in TXT/CSV formats for further processing.

### 2.1. Data Collection

The neural network algorithm for post-COVID state recognition based on ECG data was trained using a database from a clinical hospital. The database includes ECG records from 970 COVID-19 patients and over 1000 records from healthy subjects. Patients with extrasystole were excluded, but “cardiospike” anomalies were identified in COVID-19 patients’ ECG data and used to train the algorithm.

All ECG data, including the training data, are available on the COGNITOM Web platform (cogni-nn.ru) as a zip archive containing a CSV file. The CSV file includes 5 columns with the identifier, RR interval value, markup for spikes, and measurement time for each COVID-19 record. This study was conducted according to the guidelines of the Declaration of Helsinki and approved by the Ethics Committee of Lobachevsky University (Protocol number 3 from 8 April 2021).

### 2.2. Method for Determining Blood Parameters

The diagnostic blood tests were conducted at the Department of Clinical and Laboratory Diagnostics of the POMC. A biochemical blood test was performed using the “Konelab-20” automatic biochemical analyzer, manufactured by Thermo Fisher Scientific (Waltham, MA, USA). The parameters measured included ALT (U/l), AST (U/l), albumin (g/l), bilirubin unbound (indirect) (µmol/l), total bilirubin (µmol/l), bilirubin bound (direct) (µmol/l), glucose (venous) (mmol/l), blood creatinine (µmol/l), lactate dehydrogenase (LDH) (U/l), urea (mmol/l), C-reactive protein (mg/l), and ferritin (mcg/l).

Hematological studies were performed using the ABX MIKROS 60 hematological analyzer. The measured indicators included absolute basophil content ($10^{9}/l$), hematocrit (%), hemoglobin (g/l), leukocytes ($10^{9}/l$), lymphocytes (%), absolute lymphocyte content ($10^{9}/l$), monocytes (%), absolute monocyte content ($10^{9}/l$), absolute neutrophil content ($10^{9}/l$), band neutrophils (%), segmented neutrophils (%), erythrocyte sedimentation rate (mm/h), mean erythrocyte hemoglobin (pg), mean platelet volume (fl), mean erythrocyte volume (fl), mean erythrocyte hemoglobin concentration (g/l), platelet crit (%), platelets ($10^{9}/l$), erythrocyte distribution width (coefficient of variation) (%), platelet distribution width (%), absolute eosinophil content ($10^{9}/l$), and erythrocytes ($10^{12}/l$).

Hemostasis was assessed using coagulation screening indicators, which included activated partial thromboplastin time (APTT), prothrombin time (PT), fibrinogen concentration (according to Clauss), international normalized ratio (INR), and soluble fibrin monomer complexes (SFMK).

The acid–base composition of venous blood was assessed using the Rapidlab 865 blood gas analyzer, manufactured by Bayer Diagnostics, in terms of pH, electrolytes, metabolites, and oximetry. The measured indicators included absolute oxygen content O2CT (ml/dl), anion difference (mmol/l), hematocrit (%), glucose (mmol/l), deoxyhemoglobin (%), excess BE in blood (mmol/l), potassium (mmol/l), ionized calcium (mmol/l), lactate (mmol/l), sodium (mmol/l), total hemoglobin (g/l), oxyhemoglobin (%), negative logarithm of hydrogen ion concentration, oxygen partial pressure (mmHg), oxygen partial pressure at 50% saturation (mmHg), carbon dioxide partial pressure (mmHg), standard blood bicarbonate (mmol/l), functional oxygen saturation (%), and chloride level (mmol/l).

### 2.3. Machine Learning Algorithms

**Figure 2:**
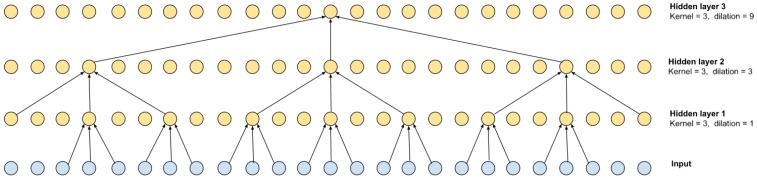
Dilated convolution with kernel = 3 and its receptive field.

To solve the problem of segmenting the flow of RR intervals, a dilated convolution network was chosen. Convolutional networks have a higher performance and learning speed than recurrent networks (RNNs), which are commonly used in time series detection and segmentation problems. Convolutional networks, on the other hand, require more convolutions to achieve a sufficient receptive field, i.e., capture long enough time sequences. To eliminate this shortcoming, stretched convolutions were used. An example for 3 layers is shown in Figure 2.

The stretch for each subsequent layer can be defined as $d=k^{n-1}$, where *n* is the number of the convolutional layer. As can be seen from the scheme shown in Figure 2, the receptive field is 27 and is defined as $r=\left(k-1\right)\sum_{i=1}^{L}k^{i-1}+1$, where *L* is the number of layers. Of course, such a multilayer scheme can be equivalent to 1 convolutional layer with a kernel of 27, but the calculation speed of the multilayer version is higher.

**Figure 3:**
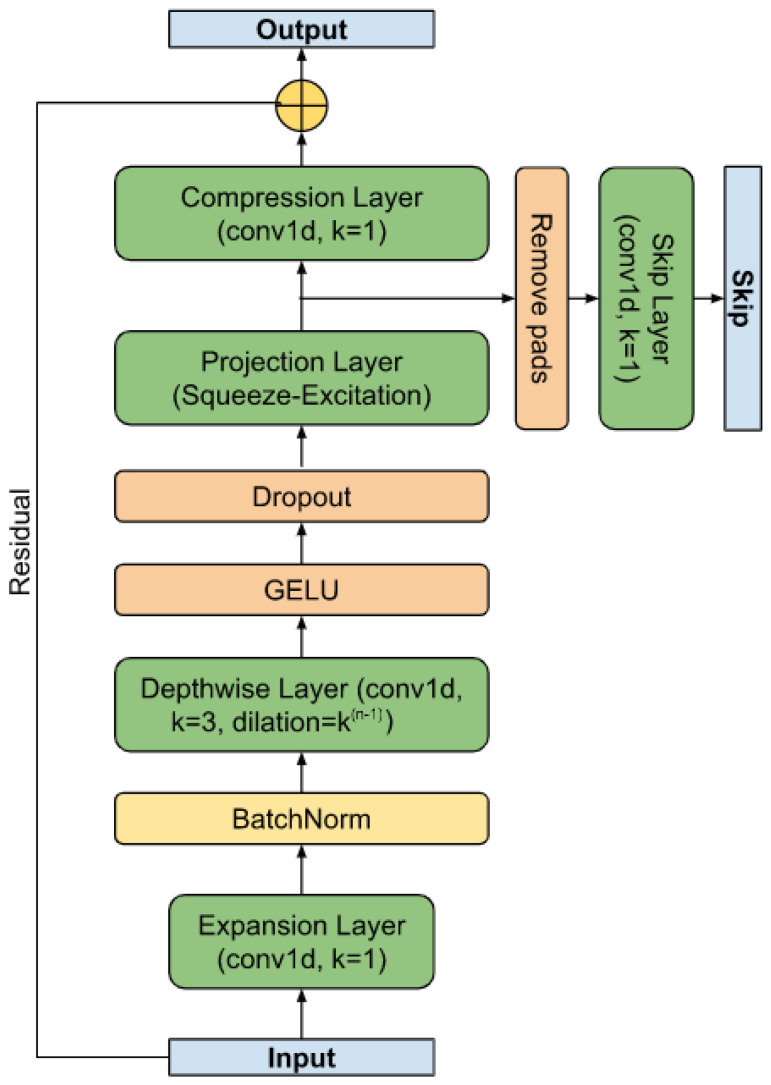
The residual block.

As shown in [58], the speed of the convolutional layer can also be increased by dividing the convolution into horizontal channel-by-channel convolutions and vertical inter-channel mixing. This scheme is borrowed for the base block of the RR interval segmenter. The residual block (see Figure 3) is a variant of the MobileNetv3 base block adapted for time series. It retains the basic principle of expanding the feature space from size (*T*, *C*) to (*T*, *H*) (expansion layer), per-channel convolution with gaps, non-linearity based on GELU [59], projection of the input (*T*, *H*) based on the Squeeze-and-Excitation approach [60], as well as a layer of inter-channel mixing to the original size (*T*, *C*) (compression layer), to the output of which the input (residual) is mixed. To speed up convergence, parameterized dimension reset (Ts, *S*) (skip) was used, which allows more deep layers to be involved in learning at the earliest stage [61]. It is worth noting that the temporal dimension of the skip output is truncated on the left and right Ts=T−2P, where *P* is the size of the padding. The value of *P* is determined by the truncation of the receptive field of the extreme elements, caused by the addition of convolutions with boundary values to preserve the dimension, as well as the maximum duration of the desired object.

**Figure 4:**
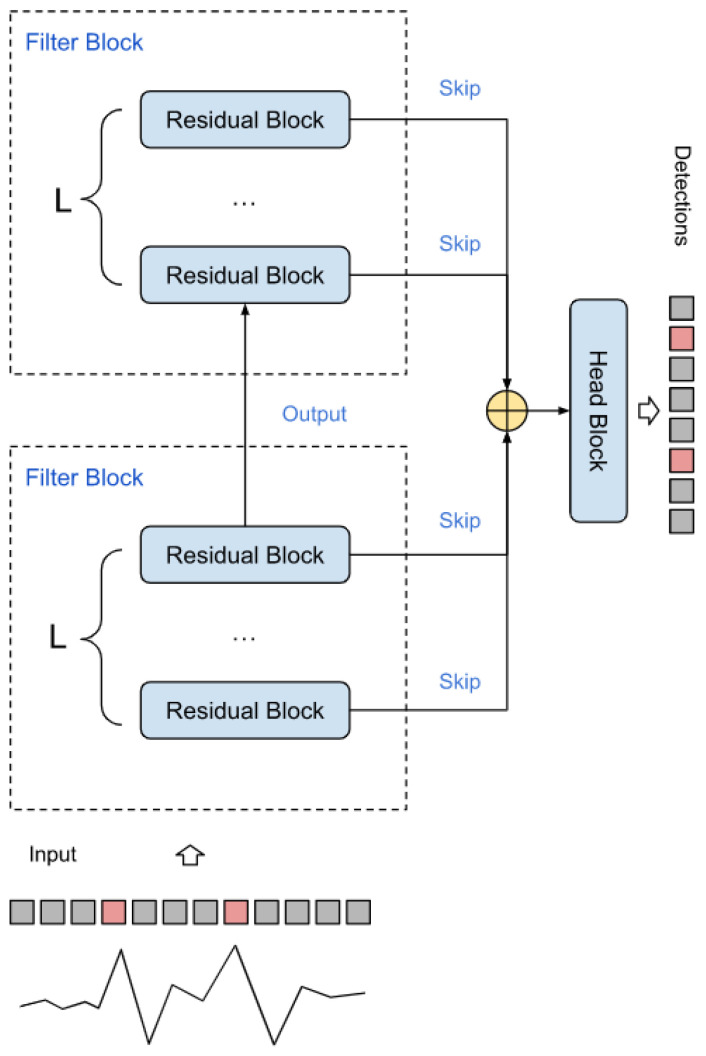
Spike detector circuit.

**Figure 5:**
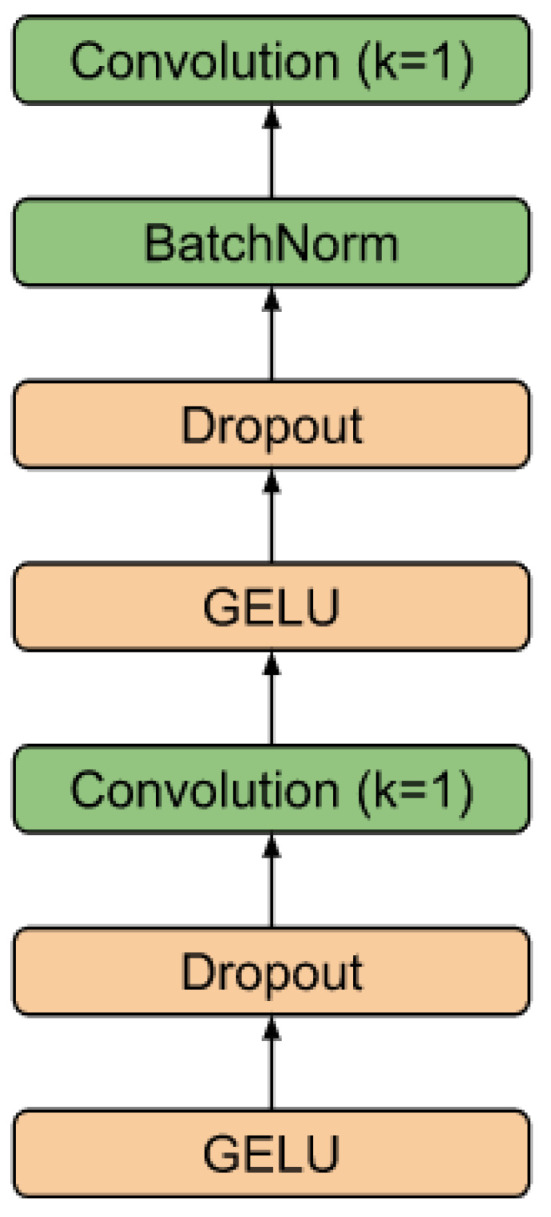
Head block.

Figure 4 shows the schematic of the RR interval detector. It consists of *F* filters, each with *L* base blocks connected in series. The skip branches of each of the layers are summed up and fed into the final segmenting unit. The diagram of this block is shown in Figure 5. The block converts the dimension input (Ts, *S*) into the output (Ts, *M*), where *M* is the number of classes (in the work M=1).

To train the model, we used labeled records of RR intervals, which are sequences of delays between adjacent QRS impulses on a cardiogram. The records are divided into segments of length *T* with an overlap of 2P; the response of the Ts model corresponds to the central part of the segment, which is T=P+Ts+P. On the records of RR intervals, the maximum values of the desired spike were previously marked, which is the goal of the study; these values unambiguously describe the position and size of the spike. The loss function was chosen based on the well-known focal loss [62]. The AdamW function [63] was used as an optimizer.

## 3. Results

### 3.1. Registration and Collection of ECG Data

**Figure 6:**
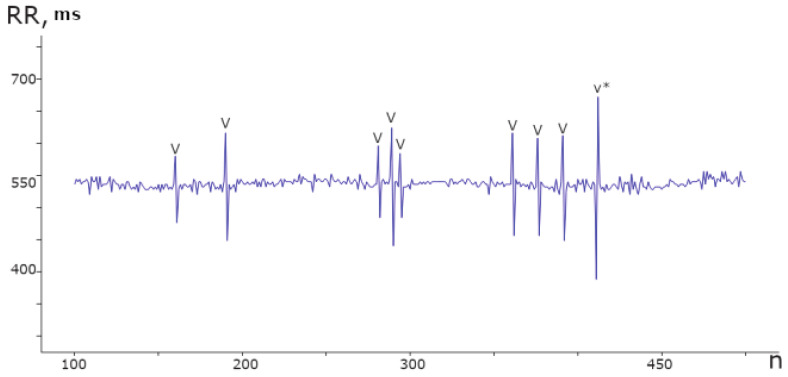
Segment of the rhythmogram of a patient with COVID-19 comprising 400 counts, displaying 9 instances of spike anomalies. These anomalies exhibit a distinct repetitive pattern and varying amplitudes within the range of ±100 ms from the mean value.

The heart, acting as a phase sensor, provides information about an organism’s functional state through rhythmograms, which are optimal signals for displaying physiological systems’ operating modes. Rhythmograms are used in various fields, including sports medicine and psychophysiology, to objectively assess functional state. In COVID-19 patients, rhythmograms are registered and processed on the Cognite web platform alongside other examinations. An experimental database was created, consisting of records from 970 patients and a total of 110,330 samples. This database revealed the rigidity [64,65] of RR intervals and the presence of cardiospikes (Figure 6), which are low-amplitude anomalies observed in patients diagnosed with COVID-19. These findings were obtained from the ZephyrSmart sensor platform.

**Figure 7:**
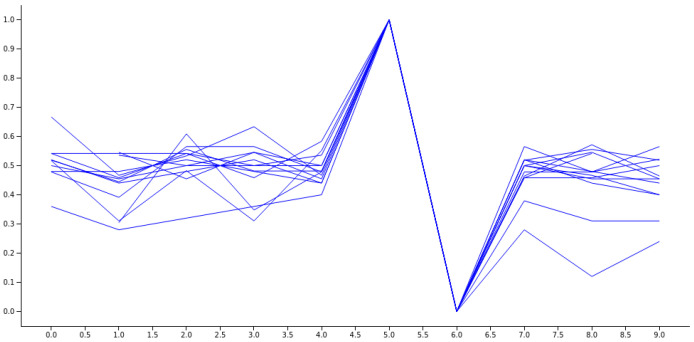
An illustration of a collection of normalized spikes from diverse subjects who have been diagnosed with COVID-19.

The spike pattern in the data differentiates two consecutive jumps, based on the RR number, from the average value. Specifically, a longer RR is followed by a shorter one, followed by a slight relaxation, as depicted in Figure 7.

### 3.2. Verification of Cardiospikes

An additional investigation was conducted to rule out the possibility that cardiospikes are linked to idiosyncrasies in signal recording and processing within the sensory platform.

**Figure 8:**
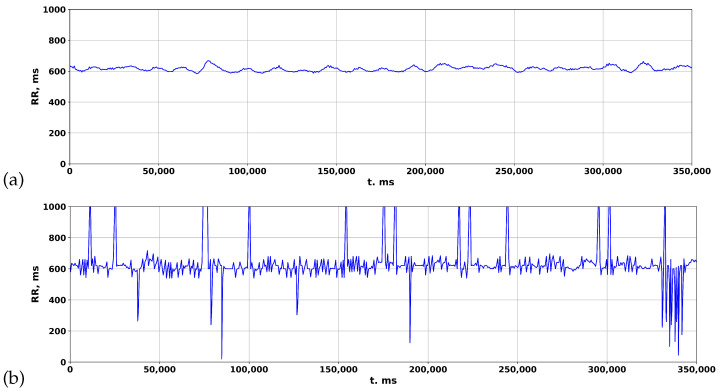
Comparing rhythmograms obtained from various devices in a patient with COVID-19 over a long-term period: (**a**) Poly-Spectrum-8; (**b**) Zephyr.

The accuracy of ZephyrSmart data was verified by comparing it with data obtained from a professional electrocardiograph, Poly-Spectrum-8 (Neurosoft). Data logging was synchronized between ZephyrSmart and Poly-Spectrum-8 based on the start time. Upon comparing the rhythmograms obtained from the two devices, it was observed that cardiospikes present in the ZephyrSmart rhythmogram were not replicated in the Poly-Spectrum-8 rhythmogram, despite a high overall correlation between the two signals (Figure 8). To simplify visual comparisons, the data are displayed on a single scale.

Based on this observation, the authors hypothesized that the algorithm for detecting QRS complexes, which includes filtering, preprocessing, and postprocessing methods, may affect the final rhythmogram signal by removing spike episodes. This hypothesis aligns with the statistical principle of heart rate variability analysis, which focuses on studying the totality of RR intervals rather than individual events. To test this hypothesis, various established algorithms for detecting R peaks in the original ECG signal were investigated.

We used algorithms from open sources [66,67]:

- Pan–Tompkins;
- Hamilton;
- Two-Moving-Average.

**Figure 9:**
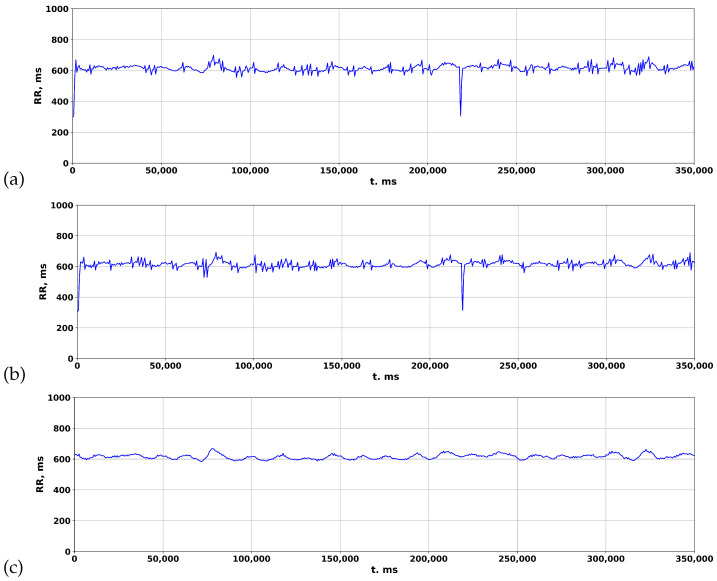
Comparison of rhythmograms in a patient with COVID-19 obtained using different algorithms: (**a**) Pan–Tompkins; (**b**) Hamilton; (**c**) Two-Moving-Average.

The rhythmogram results of processing the ECG of a patient with COVID-19 are depicted in Figure 9. In the standard package, Poly-Spectrum-8, the Two-Moving-Average algorithm is utilized to calculate rhythmograms from the ECG, which normalizes the signal and reduces deviations from the mean values (Figure 9c). However, when the Pan–Tompkins and Hamilton algorithms are used to calculate rhythmograms from the same ECG, distinctive patterns of RR intervals appear in the rhythmogram (Figure 9a,b), resembling cardiospikes observed in ZephyrSmart (refer to Figure 6).

These findings suggest that cardiospikes are not a result of hardware–software signal distortions, but rather have an endogenous nature, and may serve as markers for COVID-specific modes of heart rhythm regulation.

### 3.3. Measurement of Blood Parameters

**Figure 10:**
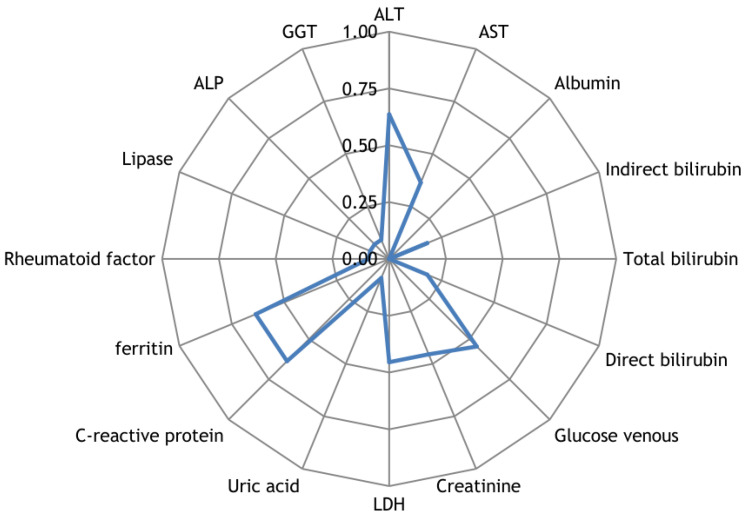
The space of parameters from the biochemistry module of indicators reflecting the state of the blood.

**Figure 11:**
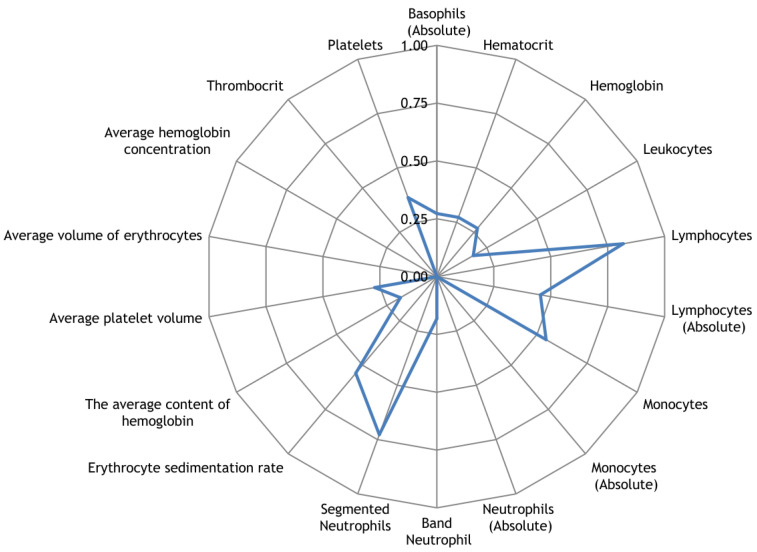
The space of parameters from the hematological studies module of indicators reflecting the state of the blood.

**Figure 12:**
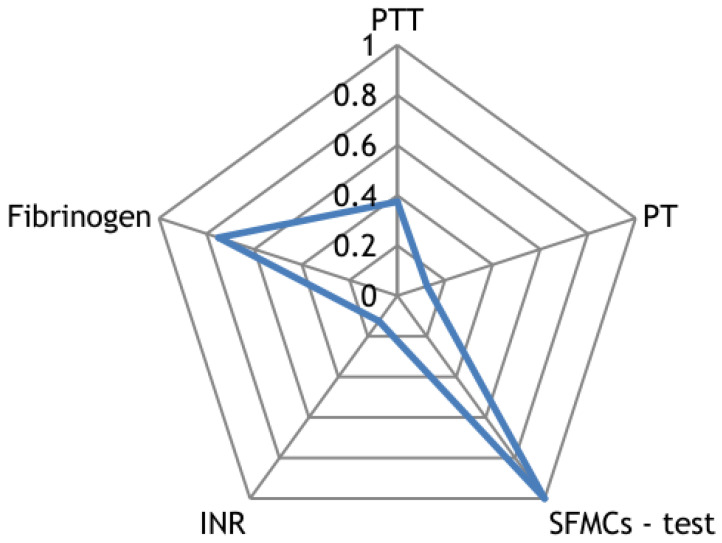
The space of parameters from the hemostasis module of indicators reflecting the state of the blood.

**Figure 13:**
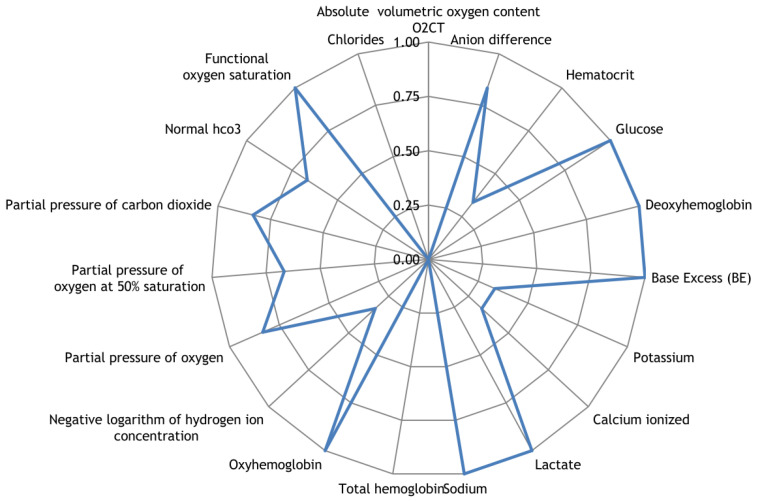
The space of parameters from the acid–base composition of venous blood module of indicators reflecting the state of the blood.

Along with the registration of RR intervals in patients with COVID-19, blood parameters were measured. To search for possible reasons for the appearance of cardiospikes, 11 patients were selected with sufficiently high frequencies of cardiospike appearance in the cardiointervalogram. We studied the space of parameters from four modules of indicators reflecting the state of the blood: biochemistry (Figure 10); hematological studies (Figure 11); hemostasis (Figure 12); acid–base composition of venous blood (Figure 13). In each module, patients were found with deviations from the normal values of the parameters under consideration. The results of the analysis are shown in the Figure 10, Figure 11, Figure 12 and Figure 13. The “biochemistry” module is distinguished by the greatest excess of normal values in terms of ALT, ferritin (0.64), C-reactive protein (0.64), and glucose (0.55). The module “hematological studies” shows the largest deviations of the following indicators: segmented neutrophils (0.73), lymphocytes (0.82), and ESR (0.55). In the module “hemostasis”, values exceed the norm for fibrinogen (0.75) and RFMC test (1). The greatest number of significant parameters occurred in the module “acid–base composition of venous blood”: glucose (1), deoxyhemoglobin (1), excess BE in the blood (1), lactate (1), sodium (1), oxyhemoglobin (1), functional oxygen saturation (1), partial pressure of oxygen and carbon dioxide (0.83), anion difference (0.83), standard blood bicarbonate (0.67).

The sample included patients with a volume of lung lesions from 44 to 92% according to the CT results. Most of them had ground glass lesions; two descriptions showed a change in transparency according to the type of fibrotic changes.

### 3.4. Data Analysis

An experimental database of records of 970 patients with a total size of 110,330 samples was collected. Of these, 2041 samples were marked as spikes. In all experiments, fixed parameters were used: sample length T=32, padding P=4, layers per block L=4, and number of blocks F=2. The choice of parameters (*H*, *C*, *S*) was based on the analysis of cross-validation experiments with the division of the database into 10 subsets. The metrics for evaluating the result were AP (average precision), $F_{1}$-score [68], and $F_{1/2}$-score. These metrics were computed from precision–recall curves, and were chosen as most closely reflecting the quality of the final model, despite the fact that each of them is insufficient at assessing the quality. As an estimate, among the 10 curves of the cross-validation sample, the worst value for the metrics was chosen.

**Figure 14:**
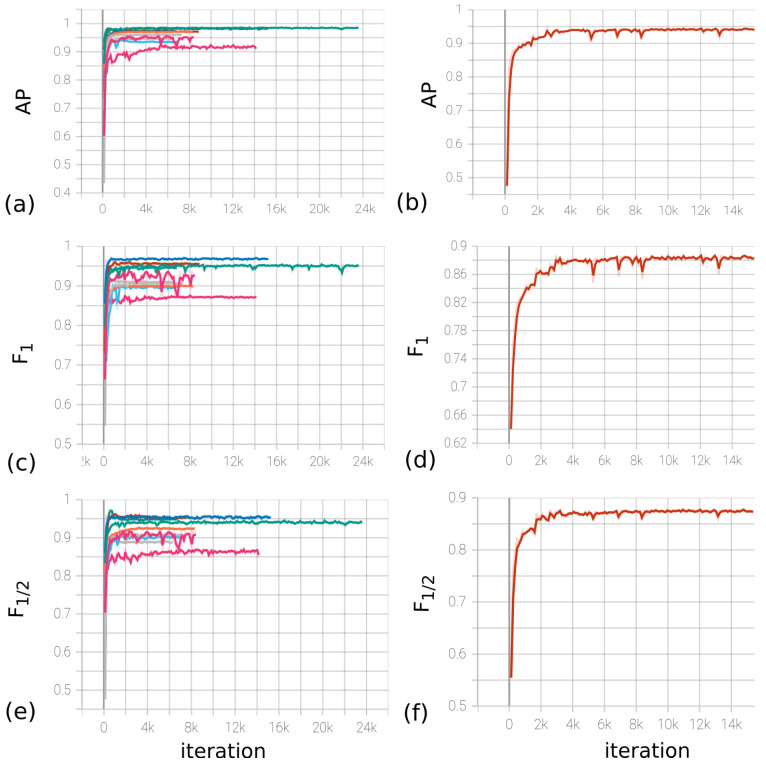
(**a**,**c**,**e**) Dynamics of metrics (from left to right, *AP*, $F_{1}$, $F_{1/2}$) depending on the iteration number during cross-validation for (*H*, *C*, *S*) = (40, 24, 72). (**b**,**d**,**f**) Dynamics of metrics (from left to right, AP, $F_{1}$, $F_{1/2}$) depending on the iteration number on the full base for (*H*, *C*, *S*) = (40, 24, 72).

Based on the measurements in Table 1, the group of model parameters (*H*, *C*, *S*) (40, 24, 72) was selected as the main parameters. The experiment results for the optimal values of the parameters are presented in Figure 14a,c,e, which display the values of the metrics depending on the gradient descent iteration.

The LR (learning rate) parameter of the optimizer was chosen to be 0.01, with the possibility of decreasing by a factor of 10 every 10 epochs. The decrease in LR occurred when the loss function for the validation set reached a plateau. The final model was trained on the division of the base into training/validation/test in the ratio 1861/180/50, respectively. Figure 14b,d,f contain the dependence of metrics on iteration. Based on the experiments, the *F*_1_-score metric on the test data indicates that the model’s accuracy is at least 0.89. Furthermore, the estimates for the model accuracy are 0.94 and 0.87, respectively.

## 4. Discussion

It is well known that disturbances in the work of the cardiovascular system and its regulation lead not only to the appearance of various heart rhythm distortions, but also to changes in the basic characteristics of the heart rate, such as heart rate variability (HRV). HRV analysis is rarely used to diagnose specific diseases. The only HRV analysis proposed by the American Heart Association for diagnostic use is the observation that low 24 h HRV in diabetes mellitus is an early sign of diabetic neuropathy [69].

The main goal of analyzing a patient’s HRV is to assess their functional state in order to evaluate the effectiveness of treatment, assess the severity of the disease, predict the risk of sudden death or dangerous complications in various diseases, and so on. The interest in HRV is primarily due to the property of the heart rhythm over time, where rigidity of a patient’s heart rhythm indicates a deterioration in their condition. R. Baevsky explains this property of the rhythm with the concept of the relationship between the adaptive capabilities of the human body and its HRV: low HRV reflects the poor adaptability of the cardiovascular system to random or permanent effects on the body [70].

In this regard, the following requirement is usually put forward for the quantitative parameters of HRV: when the patient’s condition worsens, the statistical parameters of HRV should decrease, and when the condition improves, they should increase. Since the 1960s, HRV has been studied at rest for short (up to 5 min) time intervals, while meeting strict requirements for rhythm stationarity [71].

In this case, two main characteristics of the rhythm are used: the cardiorhythmogram of rest and the magnitude of the accompanying arrhythmia. As significant parameters of HRV, both the characteristics of the histogram of the distribution of RR intervals and derivatives of these characteristics are used, including the parameters of variational pulsometry, as well as the spectral characteristics of the sequence of RR intervals. Numerous studies have shown that the deterioration of the patient’s functional state correlates with both a decrease in RR at rest and a decrease in the severity of the accompanying arrhythmia. Thus, RR at rest of 700 ms and above significantly increases the risk of cardiac death in various cardiovascular diseases, and rigidity of the resting rhythm, regardless of the value of RR, significantly increases the risk of cardiac death in myocardial infarction [72].

Since the 1980s, commercial Holter electrocardiogram (ECG) monitoring systems have been available. This made it possible to start active study of HRV over long (mainly daily) periods of time. The study of HRV in patients with various cardiovascular and other pathologies has been carried out, and many works have been published on the analysis of HRV over long periods of time. In the vast majority of these works, quantitative parameters of HRV were used, which are used in the analysis of a stationary rhythm over short periods of time [47].

It is obvious that the use of these parameters for long periods of time was effective only in the analysis of stationary (rigid or close to rigid) rhythms characterizing a very poor functional state of a person [73]. At the same time, periodic local (3–6 intervals) fluctuations in the duration of RR intervals remained outside the focus of researchers’ interests for a long time. A positive example of an in-depth clinical study of the significance of local fluctuations in the duration of RR intervals is the work of [74].

In a study by Asarcikli [75], autonomic function in the post-COVID period was examined using HRV analysis. Comparing 24 h ECG recordings of post-COVID patients with healthy controls revealed significant differences in HRV scores. Time and frequency domain indices, including SDNN 24 h, RMSSD, high frequency, and low-frequency/high-frequency ratio, were higher in post-COVID patients. The prevalence of SDNN > 60 ms and RMSSD > 40 ms was also higher among post-COVID patients. Logistic regression models supported the presence of a parasympathetic overtone in post-COVID patients, independent of covariates. These findings suggest autonomic imbalance as a potential explanation for persistent orthostatic symptoms in the post-COVID period.

The processing of medical data is essential in terms of making subsequent diagnoses and prescribing treatment or rehabilitation measures. Existing clinical systems for analyzing ECG data are based on the Two-Moving-Average algorithm, which, as our study shows, smooths the signal in such a way that significant information for diagnosing the patient’s condition disappears. However, by employing alternative algorithms, such as Pan–Tompkins and Hamilton, in the calculation of rhythmograms from ECG data, it becomes possible to acquire a more comprehensive data description.

The progress in ECG data analysis methods and their integration into clinical data analysis systems has the potential to extend the existing diagnostic capabilities for diseases, leading to significant enhancements in patients’ quality of life. Furthermore, it can facilitate the development of mobile systems for diagnosing patients’ conditions, which is a crucial undertaking in the prevention and treatment of diseases with high rates of transmission and systemic impacts on the body.

## 5. Conclusions

In this study, it was observed that patients with COVID-19 showed a distinct change in heart rate characterized by a transient increase and subsequent compensatory decrease in RR intervals. To explain the reason for such dynamics, the blood parameters of patients who had COVID-19 infection were analyzed. Based on the data obtained, it is suggested that these RR interval dynamics could be associated with profound disorders of blood coagulation and viscosity, leading to mechanical stress on the myocardium, as well as an imbalance in the acid–base balance and damage to the lung tissue. Cardiospikes, which are associated with cardiac arrhythmias in patients who had a COVID-19 infection, were found on the recorded RR intervals.

The confirmation of telemetry data using a standard electrocardiograph revealed the persistence of this phenomenon across all recording devices, emphasizing the importance of the algorithm for determining RR intervals. Importantly, the study shows that the observed spikes were not artifacts of hardware–software signal distortions, but were innate in nature, indicating their potential as markers for COVID-specific rhythm regulation regimens.

A new method for determining the post-COVID status from ECG data has been presented. By utilizing a convolutional neural network, cardiospikes present in ECG data from individuals who had a COVID-19 infection were successfully identified. The method achieved an 87 percent accuracy in detecting these cardiospikes in the test sample. These results contribute to the development of remote screening using mobile devices and heart rate telemetry for the diagnosis and monitoring of COVID-19. Further development of the study could aim at improving the accuracy of detecting cardiospikes, as well as exploring the possibility of detecting extrasystoles using the proposed method.

It should be noted that the proposed method for detecting cardiospikes was tested on data collected using the ZephyrSmart sensor platform, which allows the recording of a pure ECG signal. Other existing commercial analogues used in medical applications have built-in RR interval detection algorithms, such as the Two-Moving-Average. Our study demonstrates that such algorithms can strongly smooth the final signal by removing information about cardiospikes.

The developed method for detecting a post-COVID state based on the presence of cardiospikes in ECG recordings can serve as a valuable tool for conducting more detailed studies of the condition of patients during long-term follow-up and at various stages of the disease. This will contribute to the development of effective methods and approaches for the treatment of COVID-19.

## Figures and Tables

**Table 1:** Dependence of metrics on model parameters during cross-validation.

	*H* (Fixed C=24, S=72)			*C* (Fixed H=40, S=72)				*S* (Fixed H=40, C=24)		
**Value**	**32**	**40**	**56**	**16**	**24**	**32**	**40**	**56**	**72**	**96**
$F_{1}$	0.86	0.886	**0.89**	0.869	**0.886**	0.886	0.876	0.872	0.886	**0.89**
$F_{1/2}$	0.85	0.87	**0.891**	0.857	0.878	**0.879**	0.857	0.857	**0.87**	0.867
AP	0.93	0.943	**0.944**	0.932	**0.943**	0.934	0.93	0.929	0.943	**0.946**

## Data Availability

The data that support the findings of this study are available from the corresponding author upon reasonable request.
